# Antarctic sponges from the Terra Nova Bay (Ross Sea) host a diversified bacterial community

**DOI:** 10.1038/s41598-019-52491-0

**Published:** 2019-11-06

**Authors:** Serena Savoca, Angelina Lo Giudice, Maria Papale, Santina Mangano, Consolazione Caruso, Nunziacarla Spanò, Luigi Michaud, Carmen Rizzo

**Affiliations:** 1 Dipartimento di Scienze Chimiche, Biologiche, Farmaceutiche ed Ambientali, Universitàdi Messina, Viale F. Stagno d’Alcontres 31, 98166 Messina, Italy; 2 Istituto di Scienze Polari, Consiglio Nazionale delle Ricerche (CNR-ISP), Spianata San Raineri 86, 98122 Messina, Italy; 3 Dipartimento di Scienze Biomediche, Odontoiatriche e delle Immagini Morfologiche e Funzionali, A.O.U. Policlinico “G. Martino”, Torre Biologica, Via Consolare Valeria, 98125 Messina, Italy

**Keywords:** Microbial ecology, Biodiversity

## Abstract

Sponges represent important habitats for a community of associated (micro)organisms. Even if sponges dominate vast areas of the Antarctic shelves, few investigations have been performed on Antarctic sponge-associated bacteria. Using a culture-dependent approach, the composition of the bacterial communities associated with 14 Antarctic sponge species from different sites within the Terra Nova Bay (Ross Sea) area was analyzed. Overall, isolates were mainly affiliated to Gammaproteobacteria, followed by Actinobacteria and CF group of Bacteroidetes, being the genera *Pseudoalteromonas*, *Arthrobacter* and *Gillisia* predominant, respectively. Alphaproteobacteria and Firmicutes were less represented. Cluster analyses highlighted similarities/differences among the sponge-associated bacterial communities, also in relation to the sampling site. The gammaproteobacterial *Pseudoalteromonas* sp. SER45, *Psychrobacter* sp. SER48, and *Shewanella* sp. SER50, and the actinobacterial *Arthrobacter* sp. SER44 phylotypes occurred in association with almost all the analyzed sponge species. However, except for SER50, these phylotypes were retrieved also in seawater, indicating that they may be transient within the sponge body. The differences encountered within the bacterial communities may depend on the different sites of origin, highlighting the importance of the habitat in structuring the composition of the associated bacterial assemblages. Our data support the hypothesis of specific ecological interactions between bacteria and Porifera.

## Introduction

Sponges (phylum Porifera) are one of the most ancient extant multicellular animals and can provide valuable insights into the origin and early evolution of Metazoa^1^. As sessile filter feeders, they are capable of removing microorganisms (including bacteria, yeasts, microalgae) from the surrounding water by pumping many thousands of litres of water through their aquiferous system within the mesohyl matrix^2^. From an ecological perspective, marine sponges provide a protected and nutrient-rich niche where extensive interactions among the diverse microbial populations are fostered and probably inevitable^3–6^, allowing the establishment of microbial consortia (the overall associated community is structurally defined as a sponge holobiome) within the holobiont body^5–7^. Sponges can benefit from nutrition supply, transport of waste products and active metabolites, chemical defence against predators and biofouling, and contribution to mechanical structure^8–10^. Consequently, the microbial colonization of sponges often plays an important role in the development and evolution of the holobiont.

In this context, the extreme and remote Antarctic environment offers a unique opportunity to study the peculiar and often strict interactions that are established between Porifera, as well as other benthic hosts, and their symbionts^11^. To date, studies on the association between microbial communities and Antarctic sponges have only rarely performed [see for review^11,12^ and mainly addressed to bacterial symbionts^6,13–18^. Altogether, results highlighted that Antarctic sponge-associated bacterial communities might be sponge-specific^13,17^. Interestingly, the occurrence of the different bacterial populations inhabiting the sponge body may be inter-regulated by bacterium-bacterium interactions^6^, or intra-regulated by the production of N-Acyl homoserine lactones in the quorum sensing phenomenon^15^. Furthermore, the production of bioactive metabolites by bacterial symbionts has been demonstrated^18,19^ and it could be responsible for the selection of symbiotic bacteria.

**Figure 1:**
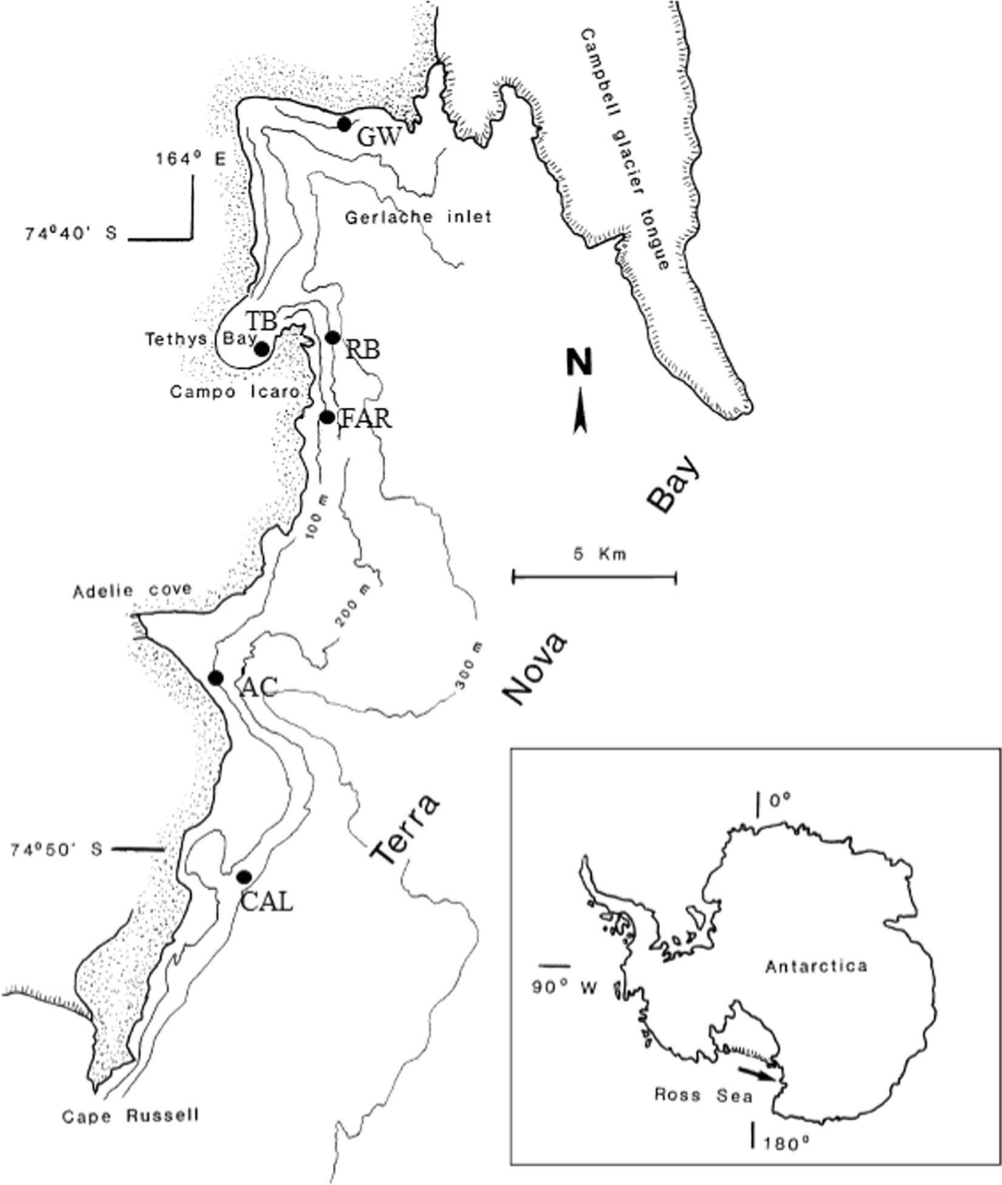
Sampling sites in the Terra Nova Bay (Ross Sea).

This study aimed at enlarging the yet scant knowledge on the bacterial communities inhabiting Antarctic sponge in particular, and Antarctic invertebrates in general^11,12^. More than 800 bacterial isolates from 14 different sponge species collected from six sites (Fig. 1) in the Terra Nova Bay area (Ross Sea) were phylogenetically identified to establish if the associated cultivable bacterial communities were host-specific or site-specific.

## Results

### Bacterial isolation and identification

In this study, a total of 595 bacterial strains were isolated from sponge specimens. To gain a more comprehensive vision on the bacterial communities associated with Antarctic sponges from the Terra Nova Bay area, obtained data were integrated with those previously obtained for sponge specimens/species collected during the same sampling period and regarding additional 290 isolates (Table 1 and reference therein). It is noteworthy that additional bacterial isolates were contextually obtained and analyzed using the identical procedure, as described in the method section.

**Table 1:** Number of bacterial strains isolated *per* sponge species.

Sponge species	Isolates (no.)	Sampling site(s)*	Reference(s)
*Anoxycalyx* (*Scolymastra*) *joubini* (Topsent, 1916)	30	TB	^6, 15^
*Calyx arcuarius* (Topsent, 1913)	62	CAL	*This study*
*Haliclona* (*Rhizoniera*) *dancoi* (Topsent, 1913)	152	CAL, GW	*This study*
*Haliclona* (*Gellius*) *rudis* (Topsent, 1901)	27	GW, TB	*This study*
*Haliclona virens *(Topsent, 1908)	41	CAL	*This study*
*Haliclona* sp.	85	TB	*This study*
*Haliclonissa verrucosa *Burton, 1932	75	AC, CAL, FAR	^18^
*Hemigellius pilosus* (Kirkpatrick, 1907)	42	CAL, GW, TB	^14^
	25		*This study*
*Lissodendoryx* (*Ectyodoryx*) *nobilis* (Ridley and Dendy, 1886)	89	AC, TB	^6, 14, 15^
*Myxodoryx hanitschi* (Kirkpatrick, 1907)	35	AC, CAL, RB	^15^
	12		*This study*
*Phorbas glaberrimus* (Topsent, 1917)	17	AC, GW, TB	^15^
	16		*This study*
*Tedania charcoti* Topsent, 1907	141	CAL, GW, TB	*This study*
	2		^16^
*Trachytedania spinata* Ridley, 1881	6	AC	*This study*
*Tedania* sp.	28	TB	*This study*
***Total***	***885***		

*AC, Adelie Cove; CAL, Caletta; FAR, Faraglioni; GW, Gondwana; RB, Road Bay; TB, Thetys Bay.

Overall, isolates mainly derived from the sponges *Haliclona dancoi* and *Tedania charcoti* (152 and 143 isolates, respectively), followed by *Lissodendoryx nobilis* (89 isolates)^6,14,15^, *Haliclona* sp. (85), *Haliclonissa verrucosa* (75)^18^ and *Calyx arcuarius* (62). Remaining sponge species yielded between 6 and 42 isolates.

Sequences from representative sponge-associated bacterial isolates were pairly aligned by BLAST^20^ to check for similarity. Isolates showing a similarity ≥97% where grouped in the same OTU/phylotype. Overall, 41 different *Alu*I ARDRA patterns (OTUs) were distinguished (Table 2 and Figure S1). Most of them (25 OTUs) were represented by 1 to 5 isolates. The cultivable bacteria were placed within five different taxa, with the predominance of Gammaproteobacteria (58%), followed by Actinobacteria (19.5%), Bacteroidetes (17.0%), Alphaproteobacteria (4.8%), and Firmicutes (0.8%). Among Gram-negative isolates (Figure S1a), Gammaproteobacteria were mainly related to the genera *Psychrobacter* (SER48 representing 80 isolates), *Pseudoalteromonas* (SER45 and SER46 representing 340 isolates), and *Shewanella* (SER50 and SER51, representing 53 isolates). Alphaproteobacteria were mainly represented by the genera *Roseovarius* (SER39 with 15 isolates), *Tateyamaria* (SER43 with nine isolates), *Sphingopyxis* (SER41 with six isolates), and *Sulfitobacter* (SER42 with five isolates). Finally, the predominant phylotype among Bacteroidetes was affiliated to the genus *Gillisia* (SER23 with 115 isolates). Among Gram-positive isolates (Figure S1b), Actinobacteria were mainly represented by *Arthrobacter*isolates (SER44 with 125 isolates), while the less represented Firmicutes included also few members in the genera *Staphylococcus* (SER33 with four isolates) and *Oceanobacillus* (SER30 with two isolates).

**Table 2:** 16S rRNA gene sequence affiliation of strains representing each Operational Taxonomic Unit (OTU) to their closest phylogenetic neighbours.

*Phylum or* Class*	OTU	Next relative by GenBank alignment (AN^a^, organism)	*Hom* ^*b*^ (*%*)	Sponge species**														
				AJ	LN	CY	HP	PG	MH	HD	HV	HR	Hsp	TC	TS	Tsp	Hver	*Total*
ALF	SER34	EF512713, *Erythrobacter* sp. JL660	99									1						*1*
	SER35	NR_102908, *Octadecabacter antarcticus* strain 307	99	1														*1*
	SER36	NR_148290, *Phaeobacter marinintestinus* strain UB-M7	97											3				*3*
	SER39	KJ475185, *Roseovarius* sp. PAMC 27236	99	1		10	1	2							1			*15*
	SER40	KX086567, *Sphingomonas* sp. UV9	100		2													*2*
	SER41	FR693315, *Sphingopyxis* sp. BB46	98	2				4										*6*
	SER42	GQ358930, *Sulfitobacter* sp. BSw21498	100	2				1								1	1	*5*
	SER43	FJ889554, *Tateyamaria* sp. KS9-11	100	3										3		1	2	*9*
GAM	SER6	LN871555, *Aliivibrio* sp. H1309/4.1	99		1			1		1				2				*5*
	SER1	AY829231, *Colwellia* sp. IE7-5	99				3		4					4		1	2	*14*
	SER2	AB085651, *Halomonas* sp. TNB I26	99			5												*5*
	SER4	CP011494, *Marinobacter psychrophilus* strain 20041	99			5										1	6	*12*
	SER45	JQ618844, *Pseudoalteromonas* sp. AECF-26b	99	6	60	9	22	2	40	9	30	3	65	76		14	1	*337*
	SER46	FJ966159, *Pseudoalteromonas* sp. BSw21424	95								3							*3*
	SER47	CP014947, *Pseudomonas koreensis* strain D26	100	1						1								*2*
	SER48	FJ785514, *Psychrobacter* sp. EB244	100	9	6	8	13	3	1	3		3	13	18		1	2	*80*
	SER3	JF721975, *Psychromonas arctica* strain HQF5	99					3										*3*
	SER50	EF628005, *Shewanella* sp. P22	99		16	7	4	5		2			1	3			8	*46*
	SER51	EU000237, *Shewanella donghaensis* KOPRI_22224	99											2			5	*7*
CFB	SER19	FR691438, *Algoriphagus antarcticus* strain R-36749	99											1				*1*
	SER20	JQ800200, *Bizionia* sp. KJF12-3	99			1	1			1		5	1			2		*11*
	SER22	AF001367, *Gelidibacter algens*	98								2					1		*3*
	SER23	NR_043125, *Gillisia hiemivivida* strain IC154	99							100		1		7		1	6	*115*
	SER27	JQ800144, *Polaribacter* sp. KJF9-6	98						1							2		*3*
	SER25	CP025117, *Olleya* sp. Bg11-27	99	4														*4*
	SER26	KT429728, *Salegentibacter* sp. R18-11	99													1		*1*
	SER28	FJ195987, *Winogradskyella* sp. NF1-39	99			8								3				*11*
ACT	SER44	JX517209, *Arthrobacter tumbae* strain C1-4c-3	99	1	4	8	17	2	1	31	4	3	2	12		1	39	*125*
	SER8	JQ680447, *Brevibacterium* sp. KS8	99									1	1					*2*
	SER9	KU560444, *Citricoccus* sp. MCCC 1A11218	99				2			1	1	6	1					*11*
	SER10	FJ795673, *Frigoribacterium* sp. 8-1	100												1			*1*
	SER11	KC469951, *Janibacter limosus* strain 1B9-2009	99							2				1	1			*4*
ACT	SER12	JX428875, *Leifsonia* sp. ZS335	99				2			1						1		*4*
	SER13	KJ475136, *Marisediminicola antarctica* PAMC 27228	99								1	2		3	1			*7*
	SER14	KX083528, *Microbacterium oxydans* strain FQ-57-1	99				2						1					*3*
	SER15	KC160891, *Mycetocola* sp. SS6.19	99									2						*2*
	SER16	CP015079, *Nocardioides dokdonensis* FR1436	99												1			*1*
	SER17	CP015235, *Rhodococcus fascians* D188	100			1		8						2	1		1	*13*
FIR	SER30	KU740186, *Oceanobacillus picturae* strain KV6	99					1									1	*2*
	SER32	JQ684228, *Planococcus antarcticus* strain HWG-A5	99					1										*1*
	SER33	LN774671, *Staphylococcus equorum* 0312MAR1A8	99											3			1	*4*
Total				*30*	*89*	*62*	*67*	*33*	*47*	*152*	*41*	*27*	*85*	*143*	*6*	*28*	*75*	885

*ALF, Alphaproteobacteria; **GAM**, Gammaproteobacteria; **CFB**, CF group of Bacteroidetes; **ACT**, Actinobacteria; **FIR**, Firmicutes. **AJ, *Anoxycalyx joubini*; **LN**, *Lissodendoryx nobilis*; **CY**, *Calyx arcuarius*; **HP**, *Hemigellius pilosus*; **PG**, *Phorbas glaberrimus*; **MH**, *Myxodoryx hanitschi*; **HD**, *Haliclona dancoi*; **HV**, *Haliclona virens*; **HR**, *Haliclona rudis*; **Hsp**, *Haliclona* sp.; **TC**, *Tedania charcoti*; **TS**, *Trachytedania spinata*; **Tsp**, *Tedania* sp.; **Hver**, *Haliclonissa verrucosa*. ^(**va**)^**AN**: Accession Number. ^(**b**)^**Hom**: sequence homology.

**Figure 2:**
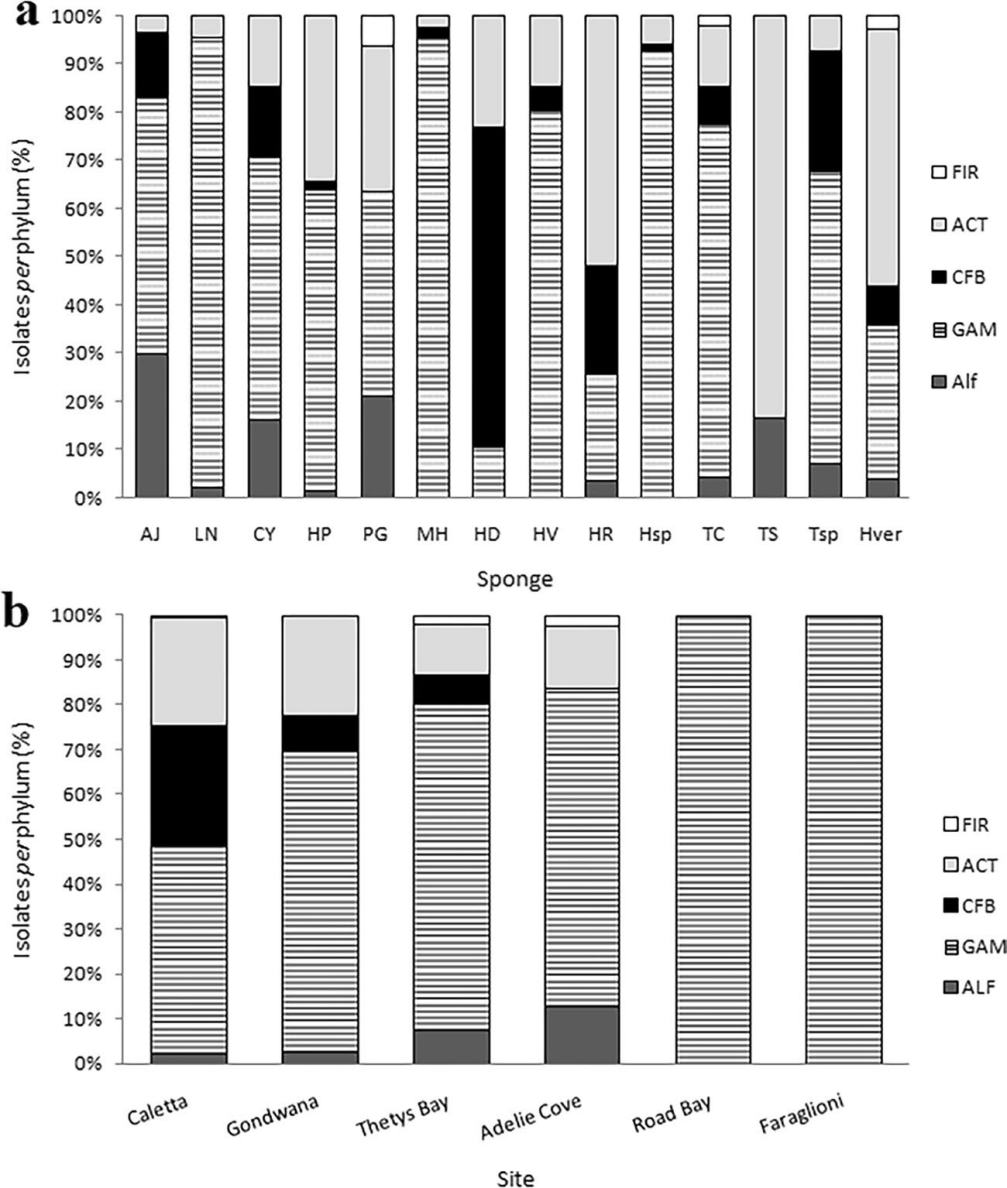
Percentage (%) incidence of each phylogenetic group in the bacterial community associated with sponges (**a**) and in different sampling sites. (**b**) **AJ**, *Anoxycalyx joubini*; **LN**, *Lissodendoryx nobilis*; **CY**, *Calyx arcuaria*; **HP**, *Hemigellius pilosus*; **PG**, *Phorbas glaberrimus*; **MH**, *Myxodoryx hanitschi*; **HD**, *Haliclona dancoi*; **HV**, *Haliclona virens*; **HR**, *Haliclona rudis*; **Hsp**, *Haliclona* sp.; **TC**, *Tedania charcoti*; **TS**, *Tedania spinata*; **Tsp**, *Tedania* sp.; **Hver**, *Haliclonissa verrucosa*. **FIR**, Firmicutes; **ACT**, Actinobacteria; **CFB**, Bacteroidetes; **GAM**, Gammaproteobacteria; **Alf**, Alphaproteobacteria.

The relative bacterial community composition reported above was generally observed for almost all sponge species. In general, Gammaproteobacteria and Actinobacteria were isolated from all sponge samples, while the other phyla appeared differently distributed among sponge species (Fig. 2a). In some cases (e.g. *Phorbas glaberrimus* and *Haliclonissa verrucosa*), Gammaproteobacteria and Actinobacteria were equally dominant, whereas Bacteroidetes dominated (149 out of 152 isolates) within the *Haliclona dancoi* bacterial community. Gammaproteobacteria constituted quite the entire bacterial community in *Lissodendoryx nobilis*, *Haliclona* sp., *Haliclona virens* and *Myxodoryx hanitschi*, whereas Actinobacteria were mainly associated with *Tedania spinata*, *Haliclona rudis* and *Haliclonissa verrucosa*. Alphaproteobacteria and Firmicutes occurred only in few sponge species.

*Tedania charcoti* was the sponge with the highest number (16) of different retrieved phylotypes, followed by *Haliclonissa verrucosa* and *Tedania* sp. (13 OTUs each). Only few phylotypes (i.e. *Pseudoalteromonas* SER45, *Psychrobacter* SER48 and *Arthrobacter* SER44) were common to almost all sponge species, whereas most OTUs were ascribed only to a single species. For instance, *Erythrobacter* SER34 and *Mycetocola* SER15 were isolated exclusively from *Haliclona rudis*, *Octadecabacter* SER35 and *Olleya* SER25 were found only in association with *Anoxycalyx joubini*, and *Sphingomonas* SER40 was retrieved only in *Lissodendoryx nobilis*.

OTU abundances resulted higher in *Tedania charcoti*, *Tedania* sp., *Haliclonissa verrucosa* and *Haliclona dancoi*, with a total of 16, 13, 13 and 11 OTUs, respectively. Bacterial community structure was compared between sponges using common ecological metrics for richness, diversity and evenness calculated for each sample, and reported in Table S1. Ecological indices showed that sponges *Calyx arcuarius* and *Phorbas glaberrimus* presented higher diversity values, but sponges *Tedania spinata* and *Tedania* sp. presented higher Chao1 values, by suggesting the presence of more rare OTUs. In relation to the surrounding aquatic bacterialcommunity, the values are all higher than mean of most sponges. One-way ANOVA showed that no significant differences occurred between sponges in terms of detected OTUs.

### Overall comparison of the associated bacterial communities at sponge species level

**Figure 3:**
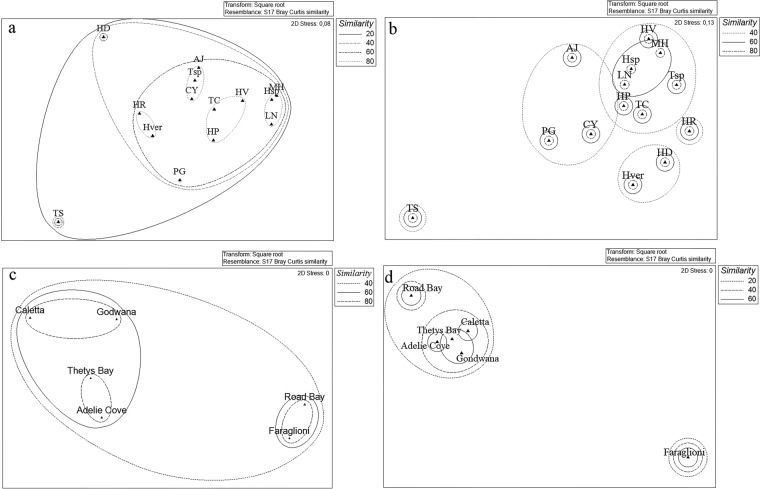
Non-metric multidimensional scaling analysis (nMDS) computed on Bray-Curtis similarity values obtained for sponges species (**a**,**b**) and sampling sites (**c**,**d**) by setting phyla (left side) and phylotypes (right side) as factors.

The cluster analysis allowed estimating similarities/differences existing between the bacterial communities associated with the different sponge species analyzed. At it is shown in Fig. 3a, using “phyla” as a factor, all bacterial communities formed a large cluster with similarity of 20%. However, *Tedania spinata* and *Haliclona dancoi* (characterized by a higher abundance of CFB group representatives) differed from the other communities, which showed a similarity of 60%. Four smaller clusters grouped sponge bacterial communities with similarity of 80%: *Lissodendoryx nobilis*, *Myxodoryx hanitschi* and *Haliclona* sp., which were characterized by a percentage of Gammaproteobacteria >90%; *Hemigellius pilosus*, *Haliclona virens* and *Tedania charcoti*, characterized by the predominance of Gammaproteobacteria ranging from 60% and 80%,and the occurrence of Actinobacteria; *Tedania* sp., *Anoxycalyx joubini* and *Calyx arcuarius* which presented a general lower relative abundance of phylum representatives; *Haliclona rudis* and *Haliclonissa verrucosa* formed the fourth cluster, characterized by a relative abundance of Actinobacteria >50%. When comparing the bacterial communities more in details using “phylotypes” as a factor, bacterial communities were grouped into two main clusters, with 20% similarity (Fig. 3b), and a strong separation of *Tedania spinata*, mainly due to the absence of *Pseudoalteromonas* sp. SER45 – which is present in all the other sponge species – and for the exclusive presence of the *Frigoribacterium* sp. SER10 and *Nocardioides* sp. SER16. The other sponge species formed four sub-clusters with similarity of 40%, among which the sponge *Haliclona rudis* grouped alone for the exclusive presence of *Erythrobacter* sp. SER34 and *Mycetocola* sp. SER15, and the higher relative abundance of *Bizionia* sp. SER20 and *Citricoccus* sp. SER9.

### Overall comparison of the associated bacterial communities at site level

With respect to the sampling site, most isolates derived from Caletta and Thetys Bay (481 and 228 isolates, respectively), followed by Adelie Cove, Gondwana and Road Bay (93, 6 and 1 isolates, respectively). Gammaproteobacteria predominated at all sites (ranging from 45.7 to 100% of total isolates *per* site) (Fig. 2b), followed by Actinobacteria (ranging from 24 to 14% of total isolates *per* site). Alphaproteobacteria were represented by 13% of total isolates at Adelie Cove, while Bacteroidetes were represented mainly at Caletta, with 26.6% of total isolates. The few isolates obtained from Road Bay and Faraglioni (six isolates and one isolate, respectively) were all affiliated to Gammaproteobacteria.

The bacterial community associated with sponges appeared different also among the sampling sites. At it is shown in Fig. 3c, using “phyla” as a factor, Thetys Bay and Adelie Cove (similar at 80%), together with Caletta and Gondwana (similar at 80%, too) grouped in a bigger cluster with 60% of similarity, while Road Bay and Faraglioni grouped in a separate cluster. A similar clustering appears when comparing the bacterial communities more in details using “phylotypes” as a factor, as well as Thetys Bay, Adelie Cove, Caletta and Gondwana formed a big cluster again with 40% similarity, while the site Faraglioni grouped alone (Fig. 3d).

The one-way ANOVA analysis showed that no difference occurred in a significantly pattern between sites, but the distribution of OTUs evidenced a significantly statistical predominance of *Pseudoalteromonas* sp. SER45 respect to all the other detected OTUs (p < 0.05).

The nMDS analysis was performed also by considering both sponge species and site (Figure S2). The ANOSIM Pairwise Test, computed by setting site as factors, showed that a significant difference between the all sites didn’t occur, while the SIMPER analysis underlined that the average dissimilarity occurring between them presented a cumulative value of 84.14% for the sites Caletta and Adelie Cove and a cumulative value of 83.38% for the sites Gondwana and Adelie Cove. The cluster analysis and nMDS were computed also on the bacterial communities of the same sponge species sampled from different sites, and on bacterial isolates from different sponge species sampled from the same site, as described below.

### OTU-sharing among sponge specimens belonging to the same sponge species, but collected from different sites

**Figure 4:**
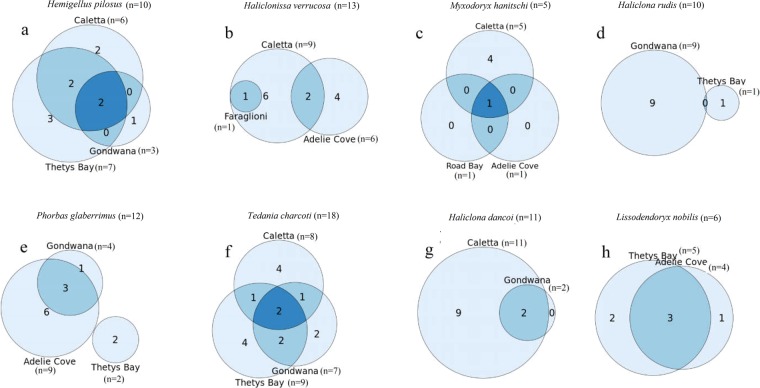
Venn diagram showing the shared and non-shared phylotypes between sponge samples collected from different sampling sites at Terra Nova Bay.

The comparison (when applicable) between the same sponge species sampled from different sampling sites at Terra Nova Bay is shown in Fig. 4.

*Hemigellius pilosus* (Fig. 4a) was obtained from the sites Caletta, Gondwana and Thetys Bay, which all shared *Pseudoalteromonas* sp. SER45and *Psychrobacter* sp. SER48, both occurring at the highest relative abundances at Gondwana (52% and 35% of total isolates, respectively). *Shewanella* sp. SER50 and *Arthrobacter* sp. SER44 were further shared between specimens collected at Caletta and Thetys Bay. The sites didn’t show significant differences in terms of OTU composition, even if Caletta presented two non-shared OTUs (i.e., *Roseovarius* sp. SER39 and *Leifsonia* sp. SER12), while Thetys Bay showed three non-shared OTUs (i.e., *Bizionia* sp. SER20, *Citricoccus* sp. SER9, and *Microbacterium* sp. SER14). It could be highlighted that the OTU *Pseudoalteromonas* sp. SER45 presented the statistical significantly higher relative abundance (p < 0.05), followed by *Arthrobacter* sp. SER44 and *Psychrobacter* sp. SER48, and then by *Shewanella* sp. SER51 and *Colwellia* sp. SER1 among sites. All the remaining OTUs occurred at relative abundances significantly lower.

*Haliclonissa verrucosa* (Fig. 4b) specimens were sampled from Caletta, Adelie Cove and Faraglioni. No significant differences among OTUs were detected in the three sites, and *Colwellia* sp. SER1 was the unique OTU that was shared between specimens collected from Caletta and Faraglioni. Conversely, *Psychrobacter* sp. SER48 and *Shewanella* sp. SER51 were shared between Caletta and Adelie Cove. Four non-shared OTUs were detected in this case at Caletta (i.e., *Arthrobacter* sp. SER44, *Rhodococcus* sp. SER17, *Staphylococcus* sp. SER33, and *Marinobacter* sp. SER4) and two at Adelie Cove (i.e., *Sulfitobacter* sp. SER42 and *Tateyamaria* sp. SER43). *Pseudoalteromonas* sp. SER45 confirmed its predominance in all the three sampling sites (i.e. Caletta, Adelie Cove and Road Bay) also in the case of *Myxodoryx hanitschi* (Fig. 4c), with a relative abundance significantly higher than the other OTUs (82%, 100%, 100%, respectively; p < 0.05). *Psychrobacter* sp. SER48, *Polaribacter* sp. SER27 and *Arthrobacter* sp. SER44 were the three non-shared OTUs among sponges collected from Caletta site.

*Haliclona rudis* (Fig. 4d) specimens didn’t share OTUs between sampling sites, while *Phorbas glaberrimus* (Fig. 4e) specimens (from Gondwana and Adelie Cove) shared *Sphingopyxis* sp. SER41, *Psychrobacter* sp. SER48 and *Shewanella* sp. SER50 (which were all absent at Thetys Bay). No significant differences occurred between OTUs and between the sampling sites in this case.

*Tedania charcoti* (Fig. 4f) specimens from Caletta, Gondwana and Thetys Bay shared *Psychrobacter* sp. SER48 and *Arthrobacter* sp. SER44, with the former that resulted at significantly higher relative abundance at the sampling sites (p < 0.05). Four non-shared OTUs were detected for Caletta (*Colwellia* sp. SER1, *Shewanella* sp. SER51, *Winogradskyella* sp. SER28, and *Rhodococcus* sp. SER17), two for Gondwana (i.e., *Aliivibrio* sp. SER6 and *Janibacter* sp. SER11), and four for Thetys Bay (Marine sponge bacterium SER36, *Algoriphagus* sp. SER19, *Marisediminicola* sp. SER13 and *Staphylococcus* sp. SER33).

*Haliclona dancoi* (Fig. 4g), sampled from Caletta and Gondwana, shared *Psychrobacter* sp. SER48 and *Pseudoalteromonas* sp. SER45, with a relative abundance of 1.4 and 3.4%, respectively, for sponge collected from Caletta, and 20% and 80% for sponge collected from Gondwana. The relative abundance of each OTU was used to check differences between specimens, but one-way ANOVA didn’t highlight significant differences in bacterial community composition with respect to phylotypes. *Aliivibrio* sp. SER6, *Pseudomonas* sp. SER47, *Shewanella* SER50, *Bizionia* sp. SER20, *Gillisia* sp. SER23, *Arthrobacter* sp. SER44, *Citricoccus* sp. SER9 and *Janibacter* sp. SER11 and *Leifsonia* sp. SER12 represented the specific OTUs detected in *Haliclona dancoi* specimens collected from Caletta.

The sites Thetys Bay and Adelie Cove shared three *Lissodendoryx nobilis* OTUs (Fig. 4h), with a relative abundance of 48 and 79% for *Pseudoalteromonas* sp. SER45, 9.1 and 5.4% for *Psychrobacter* sp. SER48, and 27 and 13% for *Shewanella* sp. SER50, respectively. In detail, *Pseudoalteromonas* sp. SER45 presented a relative abundance significantly higher than the others, followed by *Shewanella* sp. SER50, *Psychrobacter* sp. SER48 and *Arthrobacter* sp. SER44 (p < 0.05). *Aliivibrio* sp. SER6 and *Arthrobacter* sp. SER44 were the specific OTUs detected in Thetys Bay samples, while *Sphingomonas* sp. SER40 was specific for Adelie Cove.

### OTU-sharing among different sponge species collected from the same sites

**Figure 5:**
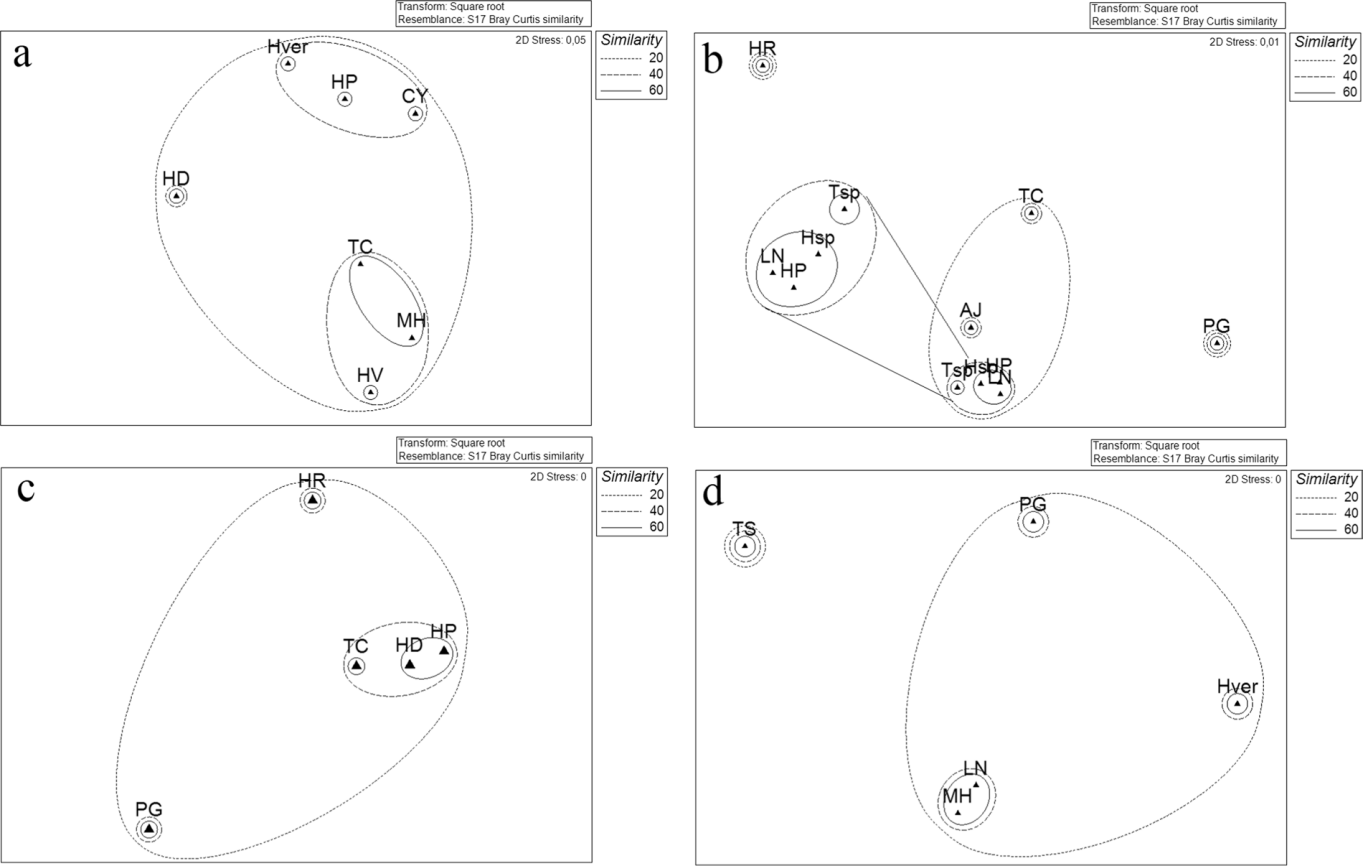
Non-metric multidimensional scaling analysis (nMDS) computed on Bray-Curtis similarity values obtained for sponges species collected from main sites: Caletta (**a**) Thetys Bay (**b**) Gondwana (**c**) Adelie Cove (**d**).

Obtained data were also analyzed (when applicable) by comparing the diversities of the bacterial communities associated with different sponge species collected from the same sites (Fig. 5).

The different sponges sampled from Caletta formed three main clusters (Bray-Curtis similarities of 20%) using phylotypes as a factor: a first cluster grouped *Calyx arcuarius*, *Hemigellius pilosus* and *Haliclonissa verrucosa*; the higher abundance of *Pseudoalteromonas* sp. SER45 probably determined the formation of a second cluster grouping *Tedania charcoti*, *Myxodoryx hanitschi* and *Haliclona virens*, while *Haliclona dancoi* grouped alone, probably due to the higher relative abundance of *Gillisia* sp. SER23 (Fig. 5a).

The Bray-Curtis similarities, calculated on the relative abundance of phylotypes associated with sponges collected from Thetys Bay, clustered together *Lyssodendoryx nobilis*, *Haliclona* sp., *Hemigellius pilosus*, with values of 60%. This was probably due to the co-occurrence of *Pseudoalteromonas* sp. SER45 and *Psychrobacter* sp. SER48. Conversely, the sponge *Phorbas glaberrimus* grouped alone due to the high relative abundance of *Arthrobacter* sp. SER44 and the exclusive presence of *Planococcus* sp. SER32 (Fig. 5b).

A similarity of 60% was shown by the sponges *Haliclona dancoi* and *Hemigellius pilosus* from Gondwana, which formed a single sub-cluster due to the comparable abundance values of *Pseudoalteromonas* sp. SER45 and *Psychrobacter* sp. SER48 (Fig. 5c).

Finally, sponges collected from Adelie Cove grouped by forming a subcluster including *Myxodoryx hanitschi* and *Lyssodendoryx nobilis* with the 60% of similarity, probably due to a higher relative abundance of the OTU *Pseudoalteromonas* sp. SER45, occurring also in *Phorbas glaberrimus* and *Haliclonissa verrucosa*, even if at lower abundances (Fig. 5d).

### Sponge bacterial community vs seawater bacterial community

Sponge-associated bacterial communities were compared to that reported by Lo Giudice *et al*.^21^ for seawater. A total of 21 phylotypes were detected in seawater and resulted absent in the sponge samples. In particular, seawater samples recorded the absolute and exclusive predominance of *Psychromonas* sp. COL1 (44% of total isolates), followed by *Pseudoalteromonas* sp. SER45(relative abundance 21%), which was shared with sponges.

**Figure 6:**
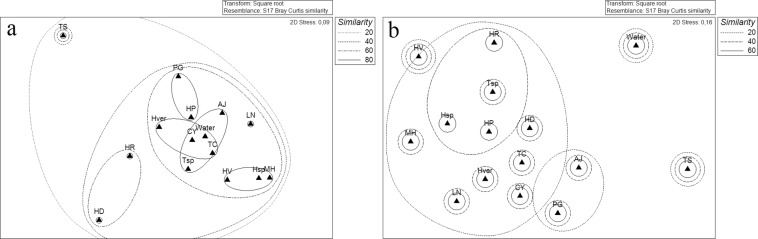
Non-metric multidimensional scaling analysis (nMDS) computed on Bray-Curtis similarity values obtained for sponges species and seawater samples by setting phyla (**a**) and phylotypes (**b**) as factors.

Overall, when using “phyla” as a factor, seawater and sponge communities showed a similarity of 80% in a cluster including *Anoxycalyx joubini*, *Tedania charcoti*, *Tedania* sp., *Calyx arcuarius* and *Haliclonissa verrucosa* (Fig. 6a). A separate cluster was formed by *Haliclona dancoi* and *Haliclona rudis*, while *Tedania spinata* grouped alone. When comparing the bacterial communities more in details using “phylotypes” as a factor, seawater community strongly differed from those observed for sponges (Fig. 6b). This separation was due to the exclusive presence of some OTUs, and for the concomitant higher abundance of common OTUs (i.e. *Sulfitobacter* sp. SER42, *Pseudoalteromonas* sp. SER45, *Polaribacter* sp. SER27, *Arthrobacter* sp. SER44, *Frigoribacterium* sp. SER10, *Leifsonia* sp. SER12, *Marisediminicola* sp. SER13, *Microbacterium* sp. SER14, *Mycetocola* sp. SER15, *Nocardioides* sp. COL46, *Rhodococcus* sp. SER17, *Oceanobacillus* sp. SER30, *Planococcus* sp. SER32 and *Staphylococcus* sp. SER33).

**Figure 7:**
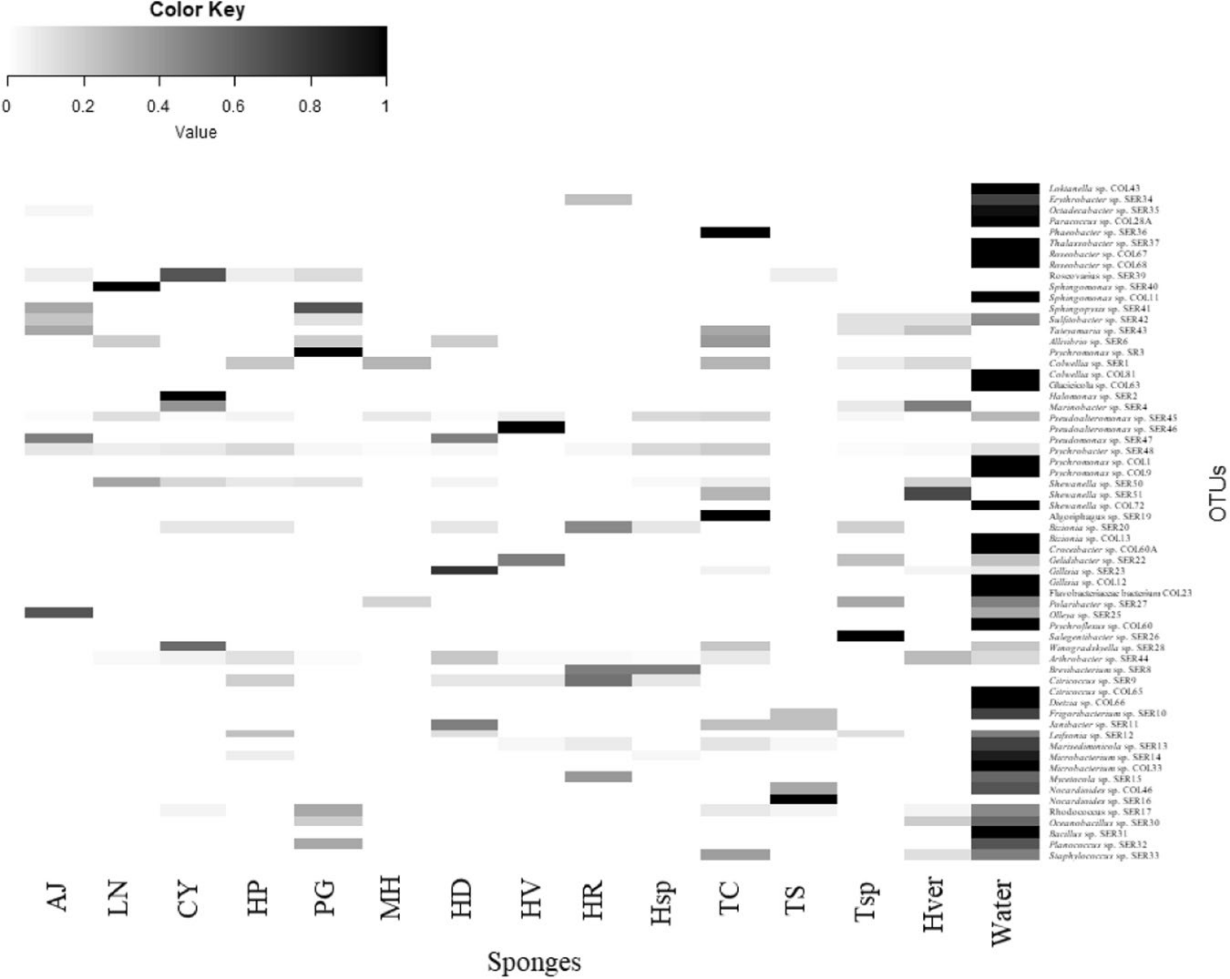
Heatmap of presence and abundance of bacterial OTUs in sponge and seawater samples. Color blocks represent the relative abundance of genera. More dark indicates a higher relative abundance.

The percentage of OTUs shared between the seawater community and at least one sponge species was about the 34% of total OTUs detected, and were more abundant than the sponge specific OTUs, as well as they together showed a relative abundance about 68%. Relative abundances of the detected OTUs and their correspondent taxonomic affiliation are shown in the heat map in Fig. 7. The picture highlighted clearly two aspects, i.e. that the most abundant OTUs among sponges and water samples were really different, and that the OTU composition among sponges was also diversified, with no groups shared among all samples. The OTUs *Pseudoalteromonas* sp. SER45 and *Psychrobacter* sp. SER48 were the only shared between all sponge samples and water (31 and 6% of total isolates, respectively). Generally, the most abundant OTUs among sponges corresponded to CFB and Actinobacteria.

## Discussion

Sponges have proven to be unique and highly selective environments to host microbial communities, often of ecological and biotechnological value^3,10,18,22–26^. In polar environments the association between microorganisms and macroinvertebrates, the physiological roles and the diversity of bacteria isolated from marine sponges are an under-investigated aspect^11^. At the time of writing, only few studies deeply characterized, through advanced sequencing techniques, the whole bacterial communities associated to different sponge species inhabiting the Antarctic coastal marine environment^13,17,27^. Similarly, few data are available concerning their cultivable fraction^6,14–16,18^. Although methodological biases could occur when applying a cultivable-based approach, bacterial cultivation could furnish complementary information to molecular-based methods in terms of community composition and it could be particularly useful in the biotechnology field in the search for novel active compounds.

Using a culture-dependent approach, we analyzed the composition of the bacterial community associated with 14 Antarctic sponge species. Overall, the bacterial community composition at phylum level (which included Proteobacteria, Bacteroidetes, Actinobacteria and Firmicutes) was similar to those previously reported by other authors^13,17,28^. Similarly, the predominance of Proteobacteria has been commonly observed within sponge-associated (analyzed by culture-dependent or culture-independent methods) bacterial communities from temperate and tropical environments^29–31^.

It is noteworthy that future experimentations are needed on Antarctic sponge-associated bacterial isolates to verify the functional diversity (which was not verified in this study) that emerged from obtained results, if considering the affiliation of retrieved bacteria. For example, Proteobacteria, generally involved in biogeochemical cycles, could have varied effects on sponge hosts, such as nitrogen fixation, sulfate-reduction function, production of low molecular-weight biological active compounds with antimicrobial and surface-active properties^3,32^. Proteobacteria were also found to produce enzymes at high levels for degrading protein and polysaccharides^33^. Among Proteobacteria, Gammaproteobacteria represented a large fraction of Antarctic sponge-associated bacterial isolates. This is not surprising, being r-strategists with the ability to rapidly grow on nutrient-rich media and successfully compete under heterotrophic conditions^21^. They were mainly affiliated to the genera *Psychrobacter*, *Pseudoalteromonas* and *Shewanella*. In particular, *Pseudoalteromonas* spp. were ubiquitarious in the analyzed samples. *Pseudoalteromonas* and *Shewanella*, isolated from both cold and temperate environments, are listed among the major producers of bioactive compounds and extracellular polymeric substances^11^, thus suggesting that they could play a pivotal role in the sponge ecology. Alphaproteobacteria isolated in this study were phylogenetically diversified, even if they were not numerically abundant (resulting 4.75% of total isolates). This is in contrast with previous investigation reporting the dominance of this taxon in association with marine invertebrates, but such result could be related to the utilization of a culture medium not suitable for their growth^34^. Among Alphaproteobacteria, members of the *Roseobacter* clade would seem to play an important role in the sulfur cycle, and the *Roseovarius* genus would be a producer of biofilms and secondary metabolites. Through the production of biofilm, they allow the adhesion to the surfaces, facilitating the colonization and acting on the ecological competition by preventing the establishment of other microorganisms^15,35^. Other alphaproteobacterial genera detected in this study, i.e. *Sulfitobacter* and *Octadecabacter*, were previously isolated from sponges^36^ and from ice and seawater, respectively^37,38^.

Following Proteobacteria, in this study Actinobacteria represented the second more abundant phylogenetic group. Actinobacterial members are well known producers of biologically active secondary metabolites, such as antibiotics or other therapeutic compounds, as well as vitamins or enzymes, that could be involved in the structuring of the bacterial community associated with Antarctic sponges^6,39–42^. It is probable that the sponges can derive different benefits from this symbiotic relationship as nutriment, degradation of substances that otherwise would not be able to dispose of and that would accumulate inside their bodies causing toxic effects on the host, as well as defense and protection thanks to the microbial production of secondary metabolites. Undoubtedly, a greater understanding of the diversity and distribution of Actinobacteria associated with sponges could contribute to the comprehension of their ecological role, in order to improve their biotechnological potential^43^. Actinobacterial isolates were affiliated to genera that were previously detected in marine or cold environments, e.g. *Arthrobacter* (which predominated in this study), *Microbacterium*, *Rhodococcus*, *Leifsonia* and *Citricoccus*^21,44–46^. Contrary to Zhang *et al*.^46^, who reported the genus *Streptomyces* as the most abundant actinobacterium isolated from sponges from temperate environments, such genus was not found among Antarctic sponge associated isolates, suggesting that environmental conditions of the sampling sites did not support its growth.

Bacteroidetes were also well represented within the Antarctic sponge-associated bacterial community. They play a key role in the carbon cycle, as they represent a group specialized in the degradation of high molecular weight compounds of the dissolved organic matter pool in the sea^47^. Among Bacteroidetes, the genus *Gillisia*, which is involved in the process of remineralization of organic matter in the ocean and is an efficient producer of secondary metabolites^48^, was particularly abundant.

The first evidence of the occurrence of Firmicutes in association with Antarctic sponges was reported by Mangano *et al*.^6^ and Papaleo *et al*.^18^ (data have been included in the present study). However, they remain scarcely represented and affiliated to the genera *Oceanobacillus*, *Planococcus* and *Staphylococcus*, all belong to non-marine bacterial groups. Firmicutes represent a fraction of the isolated microbial community also in sponges of other environments such as the Great Barrier Reef and South China Sea, which most abundant genus was represented by *Bacillus*, which has been shown to possess an efficient antibacterial activity^49^. Their involvement in biogeochemical cycles and in several degradative processes is well known^50^, therefore sponges are likely to use their metabolic products as an energy source^51^.

Even if we analyzed the sole cultivable fraction of the sponge-associated bacterial communities, a cross comparison between the different Antarctic sponge species (from the same or different sites in the Terra Nova Bay) put on evidence a possible host-specificity of associated bacteria and/or the possible influence of the sampling site. The cluster analysis showed some differences/similarities in the bacterial community composition, as previously observed by other authors^29,30^. Differences/similarities were detected mostly in terms of abundance and, in some cases, in terms of presence/absence of phylotypes. The gammaproteobacterial *Pseudoalteromonas* sp. SER45, *Psychrobacter* sp. SER48, and *Shewanella* sp. SER50, and the actinobacterial *Arthrobacter* sp. SER44 occurred in association of almost all the analyzed sponge species. However, except for *Shewanella* sp. SER50, the OTUs mentioned above were retrieved also in seawater^21^, indicating that they may be transient within the sponge body.

Accordingly to Cleary *et al*.^30^, the differences encountered within the bacterial communities may depend on the different sites of origin, highlighting the importance of the habitat in structuring the composition of the associated bacterial assemblages. The bacterial community in sponges collected from the site Caletta resulted the most abundant and diversified, and included members of great part of phyla detected, especially in the case of the sponges *Haliclona dancoi* and *Calyx arcuarius*. No bacteria isolated from Adelie Cove sponges were affiliated to Bacteroidetes, whereas Road Bay and Faraglioni specimens hosted only Gammaproteobacterial isolates at a very low percentage. However, the evident separation of the sites Road Bay and Faraglioni probably is a reflection of their scarce representativeness at level of both phyla and phylotypes. A similar bacterial community composition was observed for *Tedania charcoti*, *Myxodoryx hanitschi* and *Haliclona virens* from Caletta, *Lyssodendoryx nobilis*, *Haliclona* sp. and *Hemigellius pilosus* from Thetys Bay, *Haliclona dancoi* and *Hemigellius pilosus* from Gondwana, and *Myxodoryx hanitschi* and *Lissodendoryx nobilis* from Adelie Cove, suggesting that the site of origin could be a determinant factor in shaping the sponge associated bacterial community. At this regard, such similarity was mainly driven by the presence of *Pseudoalteromonas* SER45 and/or *Psychrobacter* SER48, which were also abundant in seawater. It could be noted, however, that a number of phylotypes (as reported in Table 2) were found only in association with a few sponge species (and often they were not present in seawater), suggesting that a close relationship between the host and the microorganisms could exist. Among them, the OTU *Shewanella* sp. SER50 presented a total abundance of 90% in the sponges, and was retrieved in eight sponges out of fourteen. While some of these phylotypes are considered quite common in Antarctic sponges, as such as *Psychromonas* and *Pseudoalteromonas*^11^, some others have been only recently reported as sponge associated genera, as it is the case of *Brevibacterium*^52^. The existence of specific microbial communities in sponges from different environments with deep differences from the surrounding waters have been previously evidenced by several authors^9,13,22,53^. Here the presence of many phylotypes detected only in sponges confirms the great potential of Antarctic sponges as *diversity reservoirs*, as just observed by Rodríguez-Marconi *et al*.^17^.

Seawater^21^ and sponge bacterial communities at Terra Nova Bay shared 21 phylotypes (out of 62), but the cluster analysis highlighted a clear separation between the two bacterial communities, which both included exclusive phylotypes or phylotypes that were particularly abundant. Our results are in line with those reported by Webster *et al*.^13^ and Rodríguez-Marconi *et al*.^17^ for McMurdo Sound and Fildes Bay, respectively, applying molecular approaches on the whole bacterial community. Taylor *et al*.^3^ suggested two main possible theories underlying the maintenance of the symbiotic relationship between bacteria and sponges, according to which microorganisms coming from the surrounding water during filtration could establish symbiosis with the host, or by vertical transmission with the reproductive processes. While in the first case a partial overlap between sponge and water bacterial community is expected, in the second one the co-evolutionary processes that would result would go to support the sponge specificity, so that a separation between sponges and water community composition occurs^29^. Our data would seem to suggest the selection by the host organism of certain taxonomic groups, supporting the hypothesis of specific ecological interactions between microorganisms and Porifera^6,15^. In order to perform a critical analysis of the bacterial community structure in terms of host-specificity, we attempted to classified each OTU as part of the *core microbiome* (i.e. a set of OTUs, absent in seawater, that were shared by at least 6 sponges), variable community member (i.e. a set of OTUs, absent in seawater, but shared by 2 to 5 sponges), or as part of the host-specific community (i.e. occurring only in a single sponge species, but absent in seawater). Based on the classification above, none of the OTUs detected in this study were part of the *core microbiome* as the most abundant (i.e. *Pseudoalteromonas* sp. SER45 and *Arthrobacter* sp. SER44) were retrieved also in seawater samples. *Shewanella* sp. SER50 presented the highest degree of specificity as it was shared by eight sponge species and was absent in seawater. The strong relation of this phylotype with Antarctic sponges could be correlated with its proven ecological role in the relationships with Porifera hosts. Several isolates affiliated to *Shewanella* spp. have been reported as involved in the production of extracellular polymeric substances, important regulators of bacterial adhesion processes, heavy metal resistance, emulsifying activities and cryoprotection^16,54^.

Differently, several OTUs (e.g. *Roseovarius* sp. SER39, *Tateyamaria* sp. SER43, *Aliivibrio* sp. SER6, *Colwellia* sp. SER1, and *Bizionia* sp. SER20) could be considered as variable community members. Finally, with regard to the host-specific community, we can speculate that *Pseudoalteromonas* sp. SER46, *Salegentibacter* sp. SER26 and *Sphingomonas* sp. SER40 may be strictly associated to the sponges *Haliclona virens*, *Tedania* sp., and *Lyssodendoryx nobilis*, respectively.

## Concluding Remarks

Although the Antarctic harsh environmental conditions make sponges a particularly attractive model for the study of symbiosis, the association between Antarctic invertebrates (not only Porifera) and microorganisms remains a very underexplored topic. Our results on the cultivable fraction of sponge-associated Antarctic bacteria suggest that, despite the ubiquitous presence of a number of phylotypes (both in sponges and seawater), some sponges could select associated bacteria. To confirm this assumption, a better characterization of the composition of the whole prokaryotic community associated with selected sponge species from the Terra Nova Bay is in progress. Even if cultivation-dependent methods have several well-known limiting factors, cultivable bacteria may represent a valuable tool in the elucidation of the main processes at the basis of sponge-bacteria interactions (e.g., bacterial adhesion, biosynthesis of molecules involved in sponge-bacteria communication and sponge bacterial selection). Further, their biotechnological value should not be underestimated, as they may represent an untapped source of novel active and useful biomolecules.

## Materials and Methods

### Sample collection

Specimens (3 to 5) of 14 Antarctic sponge species (Table 1) were collected from 6 sites at Terra Nova Bay (Ross Sea, Antarctica), namely Adelie Cove (AC; coordinates: 74°46′556″S–164°00′234″E), Caletta (CAL; coordinates: 74°45′113″S–164°05′320″E), Faraglioni (FAR; coordinates: 74°42′52″S–164°08′06.5″E), Gondwana (GW; coordinates: 74°38′00.5″S–164°09′09.8″E), Road Bay (RB; coordinates: 74°42.038′S–164°08.167′E), and Thetys Bay (TB; coordinates: 74°41.698′S–164°04′214″E) (Fig. 1). Sampling depths ranged between 25 and 200 m. Sponge specimen collection was authorized by the PRNA project. Sponge specimens were treated as previously described by Mangano *et al*.^6^. Briefly, organisms were immediately washed at least three times with filter-sterilized natural seawater to remove transient and loosely attached bacteria and/or debris. Specimens were then placed into individual sterile plastic bags containing filter-sterilized natural seawater and transported directly to the laboratory at 4 °C for microbiological processing (within 2 h after sampling). A fragment of each specimen was also preserved in 70% ethanol for taxonomic identification.

### Bacterial isolation and phylogenetic identification

Bacterial isolation from sponge was carried out as previous described by Mangano *et al*.^6^. Briefly, a central core of the sponge body was cut using an EtOH sterilized corkborer or a sterile scalpel. The sponge fragment was then aseptically weighted and manually homogenized in 0.22 µm filtered seawater in a sterile mortar. Sponge extracts were serially diluted using filter-sterilized seawater. Aliquots (100 µl) of each dilution were spread in triplicate on Marine Agar 2216 (MA, Difco). Plates were incubated in the dark at 4 °C for 1 month under aerobic conditions. Bacterial colonies grown on MA were randomly isolated and streaked at least three times before being considered pure. Cultures were routinely incubated at 4 °C. All the bacterial strains isolated from the sponges were included in the Italian Collection of Antarctic Bacteria (CIBAN) of the National Antarctic Museum (MNA) “Felice Ippolito”, and kept at the University of Messina (Italy).

#### 16S rRNA gene PCR amplification of bacterial isolates

PCR-amplification of 16S rDNA from bacterial isolates was carried out as described previously by Michaud *et al*.^55^. Briefly, a single colony of each strain was picked-up with a sterile toothpick from an MA plate, re-suspended in 20 µL of sterile distilled water and lysed by heating at 95 °C for 10 min. Cell lysates were rapidly cooled in ice and then subjected to a brief centrifugation before amplification.

Amplification of 16S rDNA was performed using the universal primer 27F (5′-AGAGTTTGATCMTGGCTCAG-3′) and 1492R (5′-TACGGYTACCTTGTTACGACTT-3′). The reaction mixtures were assembled on ice and contained 2 µL of DNA (1-10 ng DNA), 2 µL of 10X buffer, 0.6 µL of 50 mM MgCl_2_, 0.6 µL of each 10 µM forward and reverse primer (MWG, Germany), 0.4 μL of 2.5 mM dNTP mix, 0.1 µL of 5 U µL^−1^ PolyTaq polymerase (Polymed, Italy) and sterile distilled water to a final volume of 25 μL. Negative controls for DNA extraction and PCR setup (reaction mixture without a DNA template) were also used in every PCR run.

The PCR program was set as follows: (1) 95 °C for 1′30″; (2) 5 cycles at 95 °C for 30″, 60 °C for 30″ and 72 °C for 4′; (3) 5 cycles at 95 °C for 30″, 55 °C for 30″ and 72 °C for 4′; (4) 25 cycles at 95 °C for 30″, 50 °C for 30″ and 72 °C for 4′; (5) 72 °C for 10′, and a final extension step (6) 60 °C for 10′.

The amplicons were checked in an agarose gel (0.8%, w/v) in TAE buffer (0.04 M Tris-acetate, 0.001 M EDTA), containing 1 µg/ml of ethidium bromide.

#### Amplified rDNA restriction analysis

Sponge-associated bacterial isolates were grouped by the Amplified rDNA Restriction Analysis (ARDRA)^56^. Each amplicon (5 µl), containing approximately 1.5 µg of amplified 16S rDNA, were digested with 3 U of the restriction enzyme *Alu*I (Fermentas, Italy) in a total volume of 20 µl at 37 °C for 3 h. The enzyme was inactivated by heating at 65 °C for 15 min and the reaction products were analyzed by agarose (2.5%, w/v) gel electrophoresis (at 90 mV for 90 min) in TAE buffer containing 1 µg/ml of ethidium bromide^21,55^. A GeneRuler™ 100 bp DNA Ladder (Fermentas, Italy) was applied to each gel as a band reference. On the basis of the restriction patterns obtained (and visually compared one to each other), Antarctic sponge associated isolates were grouped into Operational Taxonomic Units (OTUs), assuming that one OTU was made up of strains belonging to the same species. Isolates showing identical ARDRA patterns were also checked for colony morphology on agar plates^21^.

#### 16S rRNA gene sequencing

For each OTU, one to three representative strains (where possible) were selected for sequencing by using the primer 27F. The amplicons were purified using QIAquick PCR purification KIT (Qiagen, Italy) and the subsequent sequencing was carried out at the Macrogen Laboratory (The Netherlands). Next relatives of the bacterial isolates were determined by comparison to 16S rRNA gene sequences in the NCBI GenBank and the EMBL databases using BLAST^20^. Sequences were further aligned using the program Clustal W^57^ to the most similar orthologous sequences retrieved from the database. Each alignment was checked manually and corrected. A phylogenetic tree was constructed using the MEGA X (Molecular Evolutionary Genetics Analysis) software^58^. The tree was performed using the Maximum composite Likelihood with Tajima-Nei Model (with Rate Variation and Pattern Heterogeneity) model and the Neighbour-Joining algorithm. Robustness of the inferred trees was evaluated by 1000 bootstrap re-samplings.

#### Nucleotide sequence accession numbers

Nucleotide sequences have been deposited in the GenBank database under the Accession Nos **MK660285**–**MK660324**.

### Data analyses

All statistical analyses were performed using PRIMER v6 for Windows (PRIMER-E Ltd, Plymouth, UK). Data were analyzed for eventual differences/similarities between the bacterial communities, using as a factor (**1**) the sponge species (without taking into consideration the eventual different sampling sites) or (**2**) the sampling sites (in the case of sponge specimens belonging to the same species, but collected from different sites).

Relative abundances of bacterial phyla and OTUs were opportunely transformed, and then used to calculate pairwise similarities among samples using the Bray–Curtis similarity coefficient. Bray–Curtis similarity matrices were used to perform cluster analysis and nMDS analysis of the bacterial communities from the different sponges. Analysis of Similarity (ANOSIM) was calculated to test the significance of differences among different sites using PRIMER v6 for Windows (PRIMER-E Ltd, Plymouth, UK).

Heatmaps were constructed to show the OTU distribution and clustering by using Heatplus version 2.24.0^59^ and Gplots packages version 3.0.1^60^ in R environment version 3.4.4^61,62^, using square root transformed data of relative abundance of the detected OTUs. Hierarchical clustering was generated with group average method.

The sponge-associated bacterial community composition was further compared with that previously reported by Lo Giudice *et al*.^21^ for seawater collected from the Terra Nova Bay area during the same Antarctic Expedition, and treated exactly as sponge samples for bacterial isolation. Briefly, the seawater bacterial community included 606 bacterial strains, mainly belonging to Gammaproteobacteria (68.5%; with *Psychromonas* and *Pseudoalteromonas* as predominant genera), followed by Actinobacteria (16.6%; main genera: *Microbacterium* and *Arthrobacter*), Alphaproteobacteria (9.4%; main genera: *Octadecabacter* and *Sphingomonas*), Bacteroidetes (5.8%; main genera: *Gillisia* and *Bizionia*) and Firmicutes (0.8%; main genus: *Oceanobacillus*). Each sequence from representative bacterial isolates from seawater was pairly aligned by BLAST^20^ to those obtained in this study to check for similarity. Isolates showing a similarity ≥97% where grouped in the same OTU/phylotype.

In order to evaluate the sharing or specificity level of each OTU, a threshold of presence was established and used to classify them as variable, host-specific and core sponge microbiome.

## Supplementary information

Supplementary info

## Data Availability

Raw data are available upon request.
