# Supplementary material for: Antarctic sponges from the Terra Nova Bay (Ross Sea) host a diversified bacterial community
Source: Sci Rep. 2019 Nov 6;9:16135. doi: 10.1038/s41598-019-52491-0 (PMC6834628; doi:10.1038/s41598-019-52491-0)
Supplement: Supplementary file 1 — Supplementary info [file 41598_2019_52491_MOESM1_ESM.pdf]

Supplementary materials

Manuscript Title: *Antarctic sponges from the Terra Nova Bay (Ross Sea) host a diversified bacterial community*

Authors: *Savoca Serena, Lo Giudice Angelina, Papale Maria, Mangano Santina, Caruso Consolazione, Spanò Nunziacarla, Michaud Luigi, Rizzo Carmen*

LIST

Supplementary materials

Figure S1. Neighbor-joining phylogenetic tree based on 16S rRNA gene sequences of Gram-negative strains (a) and Gram-positive strains (b) isolated from sponge samples.

Figure S2. Non-metric multidimensional scaling analysis (nMDS) computed onBray-Curtis similarity analysis conducted on the bacterial community associated with sponges for sampling site.

Table S1. Ecological indices of Antarctic sponge-associated bacterial communities compared to seawater.

a)

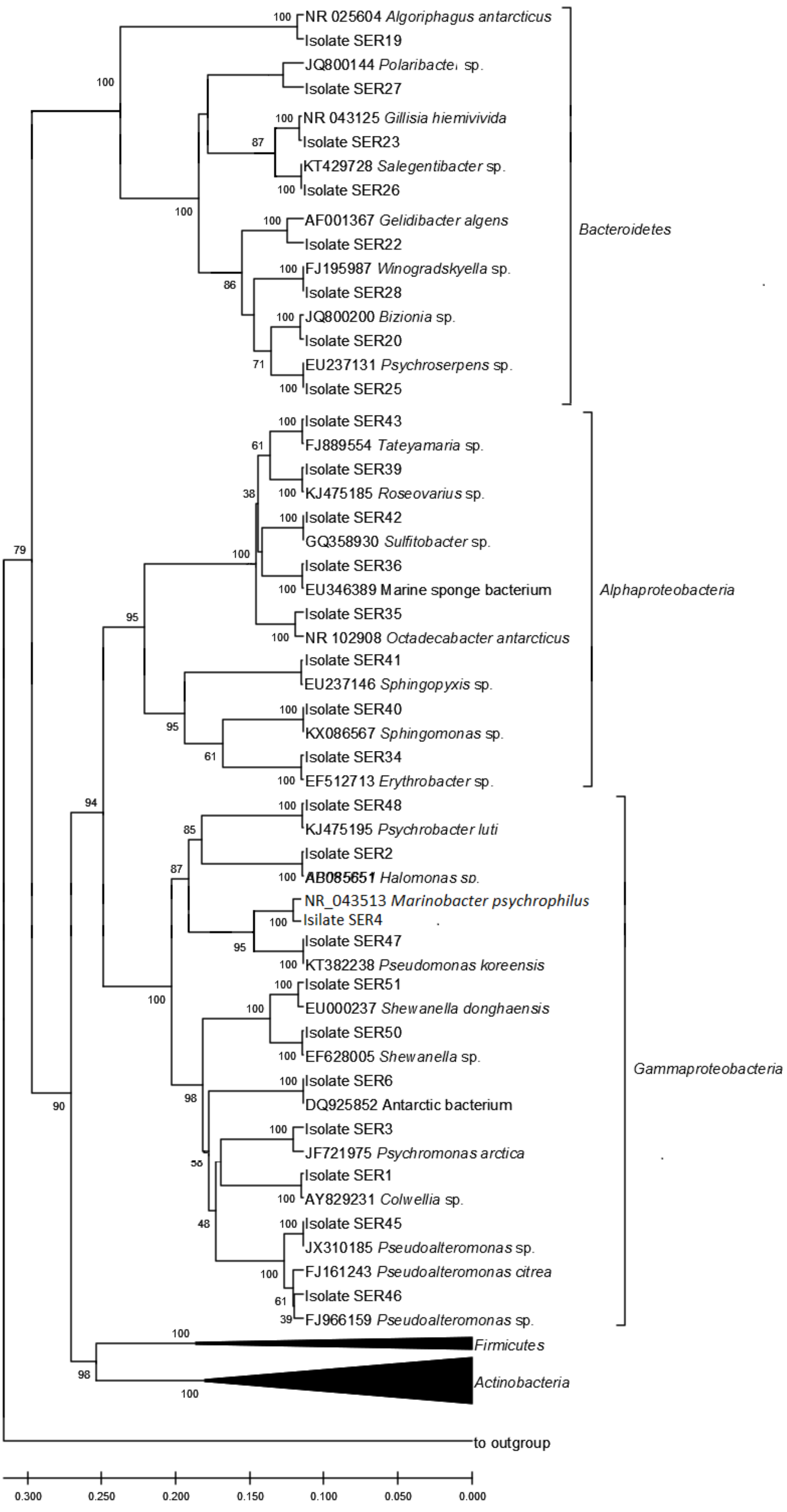
NR 025604 Algoriphagus antarcticus
Isolate SER19
JQ800144 Polaribacte sp.
Isolate SER27
NR 043125 Gillisia hiemivivida
Isolate SER23
KT429728 Salegentibacter sp.
Isolate SER26
AF001367 Gelidibacter algens
Isolate SER22
FJ195987 Winogradskyella sp.
Isolate SER28
JQ800200 Bizionia sp.
Isolate SER20
EU237131 Psychroserpens sp.
Isolate SER25
Bacteroidetes
Isolate SER43
FJ889554 Tateyamaria sp.
Isolate SER39
KJ475185 Roseovarius sp.
Isolate SER42
GQ358930 Sulfitobacter sp.
Isolate SER36
EU346389 Marine sponge bacterium
Isolate SER35
NR 102908 Octadecabacter antarcticus
Isolate SER41
EU237146 Sphingopyxis sp.
Isolate SER40
KX086567 Sphingomonas sp.
Isolate SER34
EF512713 Erythrobacter sp.
Alphaproteobacteria
Isolate SER48
KJ475195 Psychrobacter luti
Isolate SER2
AB085651 Halomonas sp.
NR_043513 Marinobacter psychrophilus
Isilate SER4
Isolate SER47
KT382238 Pseudomonas koreensis
Isolate SER51
EU000237 Shewanella donghaensis
Isolate SER50
EF628005 Shewanella sp.
Isolate SER6
DQ925852 Antarctic bacterium
Isolate SER3
JF721975 Psychromonas arctica
Isolate SER1
AY829231 Colwellia sp.
Isolate SER45
JX310185 Pseudoalteromonas sp.
FJ161243 Pseudoalteromonas citrea
Isolate SER46
FJ966159 Pseudoalteromonas sp.
Gammaproteobacteria
Firmicutes
Actinobacteria
to outgroup
0.300
0.250
0.200
0.150
0.100
0.050
0.000

b)

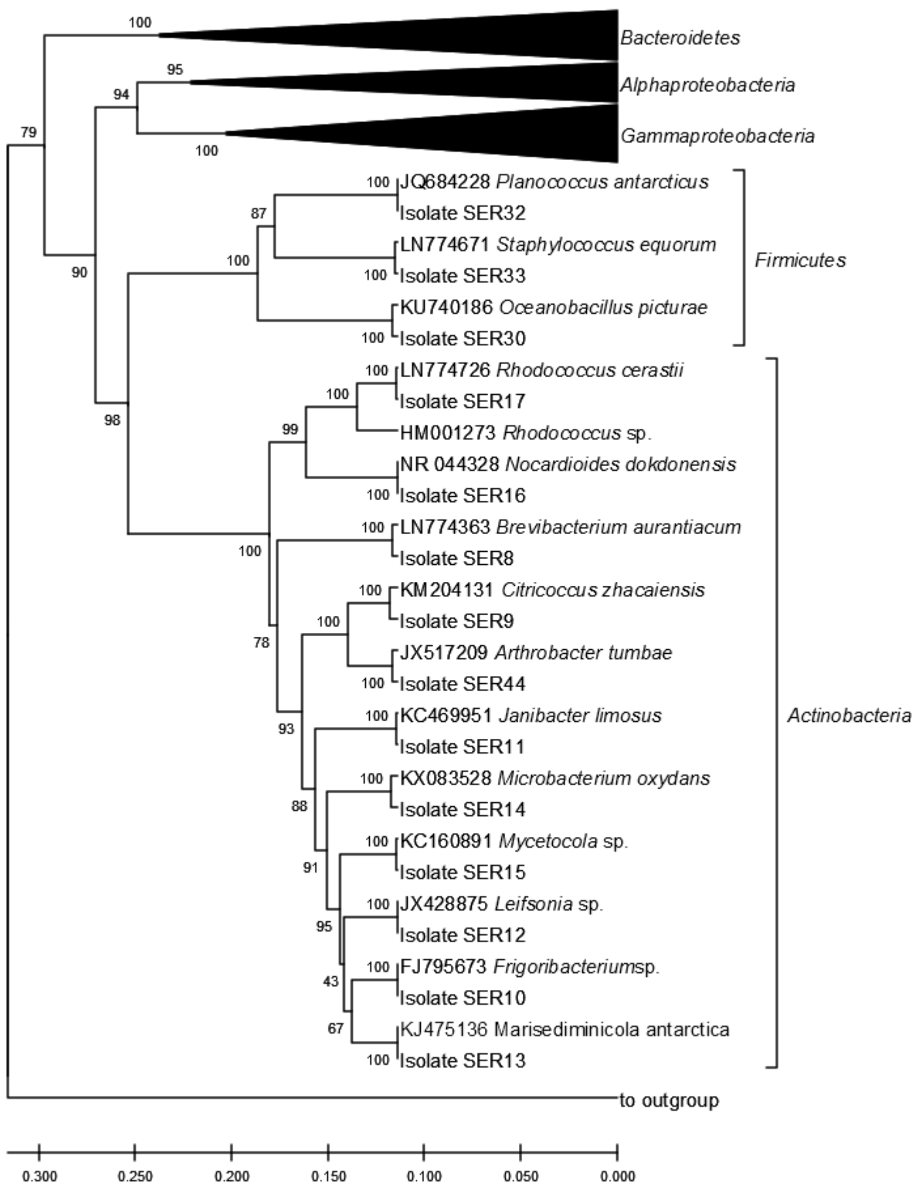

Figure S1. Savoca et al.

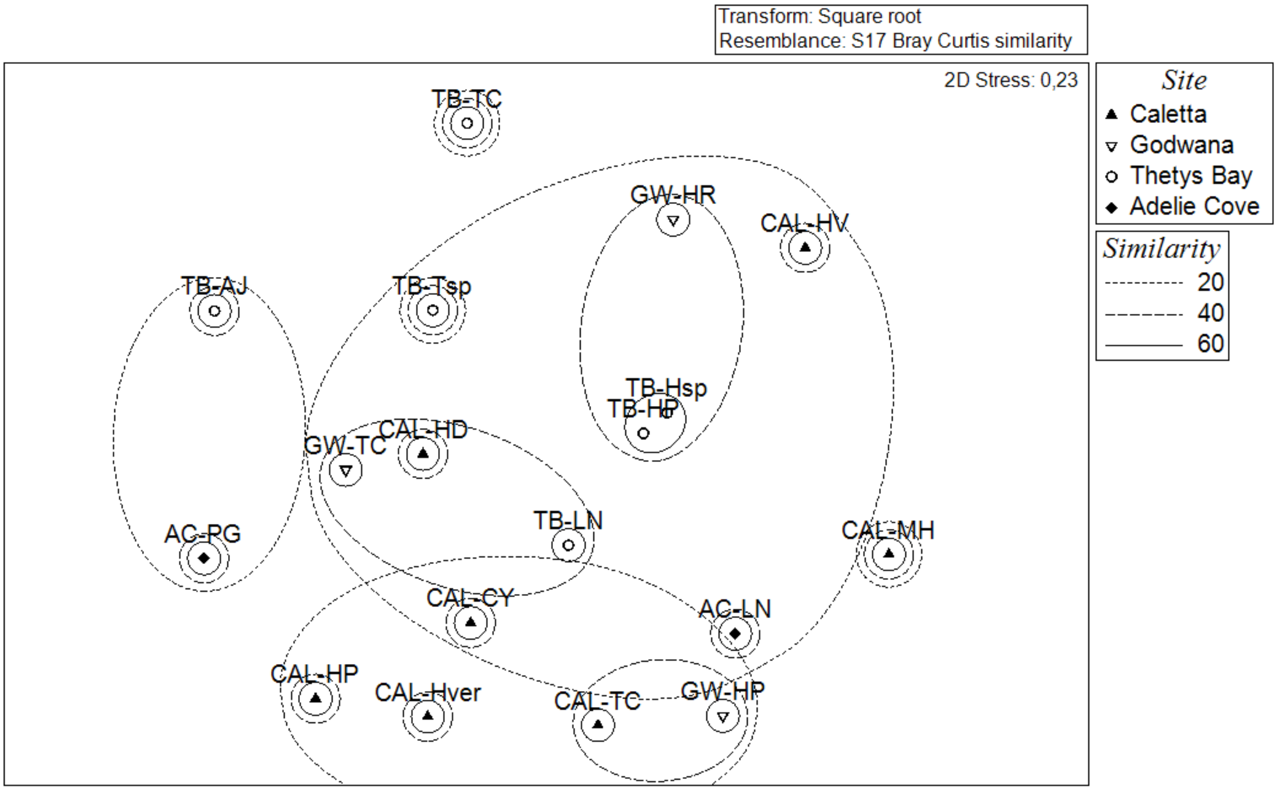

Figure S2. Savoca et al.

Table S1. Ecological indices of Antarctic sponge-associated bacterial communities compared to seawater.

Sample	OTUs			
	Taxa	Chao1	np-Shannon (H')	Simpson (D)
AJ	10.0	12.0	1.9	0.83
LN	6.0	6.0	1.03	0.51
CY	10.0	11.0	2.15	0.88
HP	10.0	10.2	1.78	0.78
PG	12.0	13.5	2.25	0.87
MH	5.0	8.0	0.59	0.27
HD	11.0	14.3	1.12	0.52
HV	6.0	6.5	0.97	0.45
HR	10.0	11.0	2.13	0.86
Hsp	8.0	13.0	0.84	0.39
TC	17.0	16.2	1.80	0.69
TS	7.0	28.0	1.95	0.86
Tsp	13.0	28.0	1.91	0.73
Hver	13.0	15.5	1.74	0.71
Seawater	*41.0*	*54.2*	*2.14*	*0.75*
